# Targeting ferroptosis, pyroptosis and necroptosis for cancer immunotherapy in melanoma: mechanistic insights and clinical perspectives

**DOI:** 10.3389/fimmu.2025.1629620

**Published:** 2025-10-29

**Authors:** Zexing Shan, Fei Liu

**Affiliations:** ^1^ Department of Gastric Surgery, Liaoning Cancer Hospital and Institute, Cancer Hospital of China Medical University, Shenyang, China; ^2^ Department of Bone and Soft Tissue Tumor Surgery, Cancer Hospital of China Medical University, Liaoning Cancer Hospital & Institute, Shenyang, China

**Keywords:** melanoma, cancer immunotherapy, ferroptosis, pyroptosis, necroptosis

## Abstract

Melanoma, an aggressive malignancy originating from melanocytes, is characterized by rapid metastasis and dismal prognosis in advanced stages, with a 5-year survival rate of only 16% for stage IV disease. Despite breakthroughs in immune checkpoint inhibitors (ICIs) targeting CTLA-4 and PD-1/PD-L1, therapeutic challenges persist, including heterogeneous response rates, acquired resistance, and immune-related toxicities, underscoring the need for strategies to augment immunogenicity and overcome immune evasion. Programmed cell death (PCD) pathways—ferroptosis, pyroptosis, and necroptosis—have emerged as critical regulators of antitumor immunity. Ferroptosis, driven by iron-dependent lipid peroxidation (LPO), enhances immunogenicity through damage-associated molecular pattern (DAMP) release and depletion of immunosuppressive cells. Pyroptosis, mediated by gasdermin (GSDM) pore formation, promotes CD8^+^ T cell infiltration via pro-inflammatory cytokine secretion, while necroptosis, governed by Receptor-Interacting Protein Kinase 1 (RIPK1)/RIPK3-MLKL signaling, facilitates antigen cross-presentation and adaptive immune memory. In melanoma, dysregulation of these pathways contributes to tumor progression and immunosuppression, yet their targeted activation reshapes the tumor microenvironment (TME) to synergize with ICIs. Current challenges, including metabolic plasticity and off-target effects, highlight the necessity for precision approaches. This review delineates the mechanistic interplay of ferroptosis, pyroptosis, and necroptosis in melanoma immunotherapy, emphasizing advances in pharmacological induction, nanotechnology-driven delivery systems, and rational combination with ICIs. By integrating preclinical insights and clinical perspectives, we propose that co-targeting these immunogenic cell death (ICD) pathways offers a transformative strategy to enhance therapeutic efficacy, circumvent resistance, and achieve durable remission in melanoma.

## 1 Introduction

Melanoma, a malignancy originating from the transformation of pigment-producing melanocytes, stands as the most aggressive and lethal form of skin cancer (1, 2). It is characterized by a high propensity for rapid metastatic spread to distant organs and, consequently, a historically high mortality rate. While early-stage, localized melanoma is often curable through surgical resection, the prognosis for patients with advanced or metastatic disease remains a formidable therapeutic challenge (3, 4). For individuals with stage IV disease, the 5-year survival rate is starkly low, estimated at approximately 16%. For decades, the therapeutic armamentarium for advanced melanoma was profoundly limited. Systemic treatments were largely confined to conventional cytotoxic chemotherapy regimens and high-dose interleukin-2 (IL-2) therapy (5, 6). These approaches, however, were plagued by significant toxicities while offering only marginal survival benefits to a very small subset of patients, leaving a critical unmet need for effective and durable treatment strategies.

**Figure 1:**
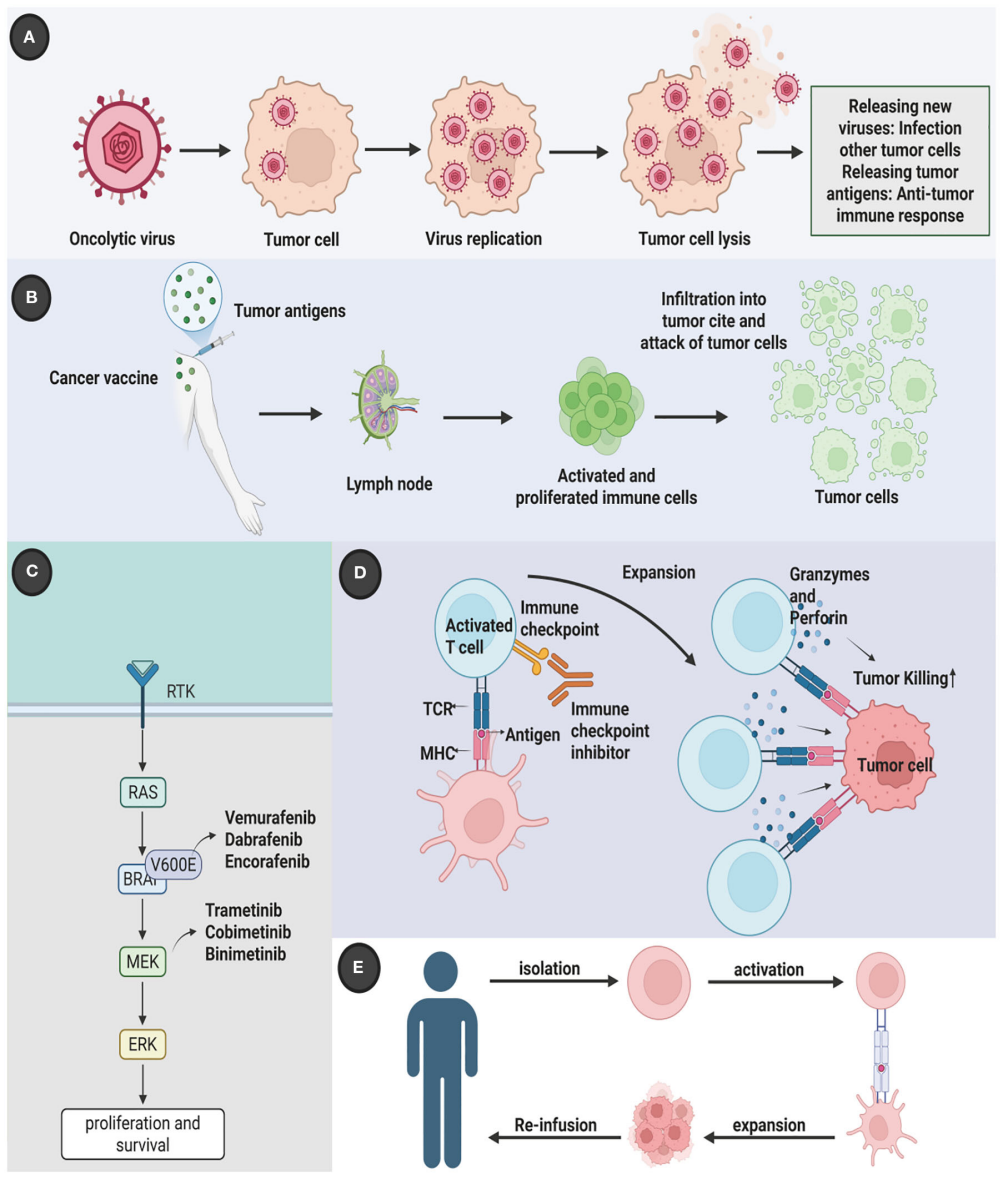
Multidimensional therapeutic innovation in melanoma: integrating molecular targeting, immune modulation, and biological precision. Contemporary melanoma management has evolved beyond conventional therapies to incorporate five strategic dimensions. **(A)** Molecular-targeted interventions counter oncogenic MAPK pathway activation through BRAF/NRAS mutation suppression; **(B)** Dual immune checkpoint blockade disrupts CTLA-4/B7-mediated T-cell anergy and PD-1/PD-L1-driven immune evasion, synergistically restoring antitumor surveillance; **(C)** Adoptive TIL therapy leverages ex vivo expanded tumor-reactive lymphocytes for precision tumor eradication; **(D)** Oncolytic virotherapy employs ICP34.5-deleted HSV-1 (T-VEC) for selective intralesional oncolysis; **(E)** Next-generation vaccine platforms coordinate cellular and nucleic acid vectors to establish durable tumor-specific immunity through effector T-cell activation and memory formation. This therapeutic matrix demonstrates enhanced tumor selectivity and reduced systemic toxicity compared to traditional cytotoxic approaches. Created by Biorender.com.

The last fifteen years have witnessed a revolutionary transformation in the management of advanced melanoma, shifting the treatment paradigm away from non-specific cytotoxic agents toward molecularly targeted and immune-based strategies. This revolution began with the advent of targeted therapy, which leverages small molecule inhibitors to block the specific driver mutations fueling cancer growth. Concurrent with these advances, a deeper understanding of tumor immunology heralded the era of immune checkpoint inhibitors (ICIs), a development that has fundamentally reshaped the prognosis for many patients. ICIs, such as anti-Cytotoxic T-Lymphocyte Antigen 4 (CTLA-4) agents (e.g., ipilimumab) and, more prominently, anti-Programmed Cell Death Protein 1 (PD-1) agents (e.g., nivolumab, pembrolizumab) and their ligands (anti-PD-L1), function by releasing the brakes on the host’s immune system, thereby reactivating cytotoxic T cells to recognize and eliminate tumor cells (7, 8) (**Figure 1**). Melanoma’s inherent immunogenicity, characterized by a high tumor mutational burden (TMB) often driven by ultraviolet (UV) radiation-induced DNA damage, makes it particularly susceptible to this class of therapy. The clinical success of ICIs has been unprecedented, with combination therapies such as ipilimumab plus nivolumab achieving durable, long-term survival in a significant portion of patients with advanced disease (9, 10). Adoptive cell therapy, which involves the infusion of genetically modified or expanded tumor-specific T cells, has shown promise, particularly in cases of refractory disease (11, 12). Oncolytic virus therapy uses genetically modified viruses to selectively infect and kill tumor cells while stimulating an immune response against the tumor (13–15). Cancer vaccines, designed to stimulate the immune system against melanoma-specific antigens, are another area of active investigation (16, 17).

Despite this remarkable progress, significant challenges persist. A substantial proportion of patients either exhibit primary resistance to ICIs or develop acquired resistance over time, leading to eventual disease progression. Moreover, the clinical utility of ICIs is often constrained by immune-related adverse events (irAEs), which can affect any organ system and range from mild to life-threatening (18, 19). Addressing these hurdles requires a shift towards greater therapeutic personalization, which hinges on the identification and validation of robust predictive biomarkers. Existing biomarkers, such as PD-L1 expression and TMB, have proven to have limited predictive power and are not consistently reliable for guiding individual patient decisions. Consequently, intensive research efforts are focused on discovering novel biomarkers. Emerging candidates in 2025 include non-invasive, blood-based markers like circulating tumor DNA (ctDNA), which can provide real-time insights into tumor dynamics and response. Furthermore, deep interrogation of the tumor microenvironment has revealed that the presence of organized immune aggregates known as tertiary lymphoid structures (TLS) is strongly associated with favorable responses to immunotherapy, positioning TLS as a highly promising tissue-based biomarker. Other innovative approaches include analyzing circulating immune cell metabolic signatures and leveraging artificial intelligence to integrate multi-omics data for more accurate response prediction. Understanding these mechanisms of immune evasion and identifying reliable biomarkers are paramount to overcoming resistance, optimizing patient selection, and developing the next generation of therapies and combinations.

Ferroptosis, pyroptosis, and necroptosis have emerged as key regulators of tumor progression and antitumor immunity. Ferroptosis, driven by iron-dependent lipid peroxidation (LPO), enhances immunogenicity by exposing calreticulin and ATP, which promote antigen presentation and the activation of immune cells. Pyroptosis, mediated by gasdermin (GSDM) pore formation, triggers the release of pro-inflammatory cytokines, facilitating CD8+ T cell infiltration and amplifying Th1 immune responses (20–22). Necroptosis, initiated by Receptor-Interacting Protein Kinase 1 (RIPK1)/RIPK3-MLKL signaling, leads to the release of High Mobility Group Box 1 (HMGB1) and supports cross-presentation of tumor antigens, thus linking cell death to immune surveillance. In melanoma, these pathways are frequently dysregulated, with ferroptosis and pyroptosis playing a role in tumor suppression, while necroptosis may contribute to chronic inflammation and immune evasion. The ability of these lytic forms of programmed cell death (PCD) to modulate immune responses highlights their therapeutic potential in cancer immunotherapy (23, 24). By inducing immunogenic cell death (ICD), ferroptosis, pyroptosis, and necroptosis can reshape the tumor microenvironment (TME) to enhance the efficacy of immune checkpoint inhibitors (ICIs). For example, ferroptosis can deplete immunosuppressive regulatory T cells (Tregs) and myeloid-derived suppressor cells (MDSCs), while pyroptosis recruits neutrophils and activates macrophages, transforming immunologically “cold” tumors into inflamed niches (24–27). Necroptosis further promotes antigen spreading and the formation of durable T cell memory. Despite preclinical advances, challenges remain in translating these findings to the clinic due to issues such as off-target toxicity, tumor metabolic plasticity, and the need for predictive biomarkers (28, 29). However, future strategies that combine Regulated Cell Death (RCD) inducers with immune checkpoint blockade (ICB) and use innovative technologies, such as tumor-penetrating nanocarriers, hold great promise for overcoming resistance and improving therapeutic outcomes in melanoma.

Overall, this review explores how the strategic targeting of ferroptosis, pyroptosis, and necroptosis can redefine melanoma immunotherapy, translating mechanistic insights into actionable clinical strategies aimed at achieving durable remissions. By bridging cutting-edge research on ICD with innovative therapeutic approaches, we anticipate a future where combinatory therapies targeting these death pathways will enhance the efficacy of immunotherapies, offering new hope for melanoma patients (30, 31).

## 2 Overview of ferroptosis, necroptosis, and pyroptosis

### 2.1 Overview of ferroptosis

Ferroptosis is an iron-dependent form of RCD driven by excessive LPO, leading to plasma membrane rupture (32–35). Key molecular mechanisms involve iron accumulation, mediated by transferrin receptor (TFRC)-dependent iron uptake and autophagy-driven degradation of iron storage proteins (36–38), which elevate intracellular labile iron pools. This iron catalyzes the production of reactive oxygen species (ROS) through Fenton reactions and activates enzymes like arachidonate lipoxygenases (ALOXs) (39–41). These enzymes oxidize polyunsaturated fatty acids (PUFAs) into phospholipid hydroperoxides (PL-PUFA-OOH) via Acyl-CoA Synthetase Long Chain Family Member 4 (ACSL4) and Lysophosphatidylcholine Acyltransferase 3 (LPCAT3), culminating in membrane damage (42–50). The glutathione GSH peroxidase 4 (Gpx4) antioxidant axis is central to ferroptosis suppression: system Xc^-^ imports cysteine for GSH synthesis, while GPX4 neutralizes lipid peroxides (51–59). Dysregulation of this system—through oncogenic pathways or p53-mediated inhibition of SLC7A11—renders cells susceptible to ferroptosis (60–66). Autophagy further amplifies ferroptosis by degrading GPX4, lipid droplets, and iron storage proteins, highlighting its role as a metabolic vulnerability in cancer (67–71).

**Figure 2:**
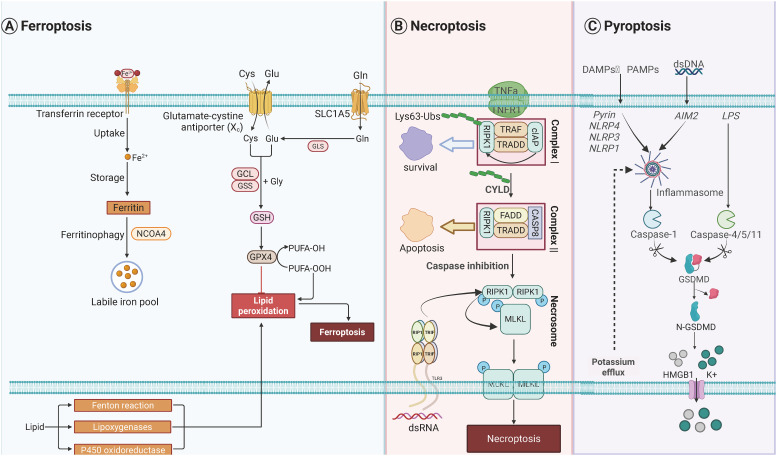
Molecular orchestrations governing three distinct programmed cell death modalities. **(A)** Pyroptotic signaling pathways are initiated through pattern recognition receptors including NLR family sensors (NLRP1/3/4), AIM2 detectors, and Pyrin proteins upon detecting pathogenic or danger signals. These sensors nucleate ASC-mediated inflammasome assembly, facilitating caspase-1 activation. Non-canonical pathway activation occurs through cytoplasmic LPS recognition by inflammatory caspases (CASP4/5/11). Both pathways converge on gasdermin-D proteolysis, generating pore-forming N-terminal fragments that execute lytic cell death. Concomitant potassium ion efflux during membrane permeabilization promotes HMGB1 liberation and extracellular potassium elevation. **(B)** Ferroptotic cell demise arises from dual metabolic dysregulation: iron homeostasis disruption and peroxidative membrane damage. Intracellular iron overload is mediated through enhanced TFRC-mediated uptake, impaired ferroportin export, and ferritin degradation via autophagy-lysosomal pathways. Lipid peroxidation cascades are driven by ACSL4-LPCAT3 enzymatic coupling facilitating ALOX-mediated polyunsaturated fatty acid oxidation, with RAB7A-regulated lipid droplet autophagy supplying peroxidation substrates. Cellular defenses against oxidative collapse include GPX4-glutathione redox cycling, AIFM2-CoQ10 antioxidant pairs, GTP cyclohydrolase-dependent radical scavenging systems, and ESCRT-III-mediated membrane repair mechanisms. **(C)** Necroptotic execution follows TNF receptor engagement and sequential signaling complex assembly. Initial membrane-proximal Complex I formation involves cIAP-mediated RIPK1 K63-ubiquitination, promoting NF-κB survival signaling. CYLD-dependent deubiquitination triggers cytosolic Complex II formation, where caspase-8 inhibition redirects signaling to RIPK1-RIPK3-MLKL necrosome assembly. Sequential kinase activation culminates in MLKL phosphorylation-induced oligomerization, forming plasma membrane-disrupting pores that mediate lytic cell death. Created by Biorender.com.

Emerging evidence highlights ferroptosis as a pivotal regulator of antitumor immunity, offering novel strategies to overcome therapeutic resistance. In pancreatic ductal adenocarcinoma (PDAC), circTRIP12 (cTRIP12) drives ferroptosis resistance by binding O-GlcNAc transferase (OGT), elevating O-GlcNAcylation to stabilize ferritin heavy chain (FTH) and PD-L1, thereby suppressing ICD and promoting immune evasion (72). Similarly, Kelch-like ECH-associated protein 1 (KEAP1)-mutant tumors exhibit ferroptosis resistance via NRF2-mediated upregulation of NQO1, which attenuates LPO and dampens immunotherapy responses; targeting NQO1 restores ferroptosis sensitivity and triggers antitumor immunity (73). Advances in nanotechnology further amplify ferroptosis-immunotherapy synergy. For small cell lung cancer (SCLC), a cationic liposome co-delivering paclitaxel and PFKFB4-targeting siRNA induces ferroptosis, reprograms the immunosuppressive microenvironment, and enhances PD-L1 blockade efficacy (74–76). In glioblastoma (GBM), magnetic exosomes co-loaded with arsenic trioxide and IR780 disrupt redox balance, augmenting photodynamic therapy to activate ferroptosis, polarize macrophages, and reinvigorate T cell responses (77–81). Hepatocellular carcinoma (HCC) studies demonstrate that lactate depletion via engineered nanoparticles sensitizes tumors to erastin-induced ferroptosis, while Fe²^+^/Fe³^+^-mediated chemodynamic therapy synergizes with αPD-L1 to suppress metastasis (82–85). Collectively, these findings underscore the dual role of ferroptosis in directly killing tumor cells and indirectly remodeling immune landscapes, with targeted delivery systems and metabolic interventions bridging mechanistic insights to clinical translation. Molecular orchestrations governing of ferroptosis was shown in **Figure 2A**.

### 2.2 Overview of necroptosis

Necroptosis represents a caspase-independent, lytic form of regulated cell death (RCD). It is primarily initiated by the activation of death receptors or pathogen-sensing receptors, serving as a critical cellular response pathway (86, 87). A well-characterized trigger of necroptosis is the binding of tumor necrosis factor-α (TNF-α) to its cognate receptor. Upon this interaction, receptor-interacting serine/threonine-protein kinase 1 (RIPK1) undergoes recruitment of RIPK3, leading to the formation of a multiprotein complex termed the necrosome (88–93). The necrosome then mediates the phosphorylation of mixed-lineage kinase domain-like protein (MLKL); once phosphorylated, MLKL undergoes oligomerization and translocates to the plasma membrane. At the membrane, oligomerized MLKL forms transmembrane pores, which disrupt membrane integrity and drive the release of damage-associated molecular patterns (DAMPs)—a hallmark of lytic cell death (94–100). This process is often triggered when apoptosis is inhibited, serving as a backup defense against pathogens. Necroptosis plays dual roles in cancer: it acts as a tumor suppressor in colorectal and hepatocellular carcinomas (101–107), where RIPK3/MLKL downregulation correlates with poor prognosis, but may promote inflammation-driven malignancy in specific contexts (108–113). Epigenetic silencing of RIPK3 in tumors can be reversed by hypomethylating agents, restoring chemosensitivity (114, 115). Key regulators include Cylindromatosis (CYLD) (116–119), which deubiquitinates RIPK1 to facilitate necrosome assembly, and Z-DNA Binding Protein 1 (ZBP1), which senses viral RNA/DNA to activate RIPK3-MLKL signaling independently of death receptors (120, 121).

Necroptosis has emerged as a potent ICD pathway to amplify antitumor immunity. In PDAC, MLKL-driven necroptosis recruit macrophages, upregulates CD47 to evade phagocytosis, and triggers CXCL8-mediated epithelial-mesenchymal transition (EMT) and liver metastasis. Combining MLKL inhibitor GW806742X with CD47 blockade suppresses metastatic dissemination, highlighting necroptosis as a dual-edged sword in PDAC progression (122–128). To leverage its immunogenicity, nanotechnology platforms have been designed to induce tumor-specific necroptosis. For instance, a sulfate radical (SO4·−)-based *in situ* vaccine triggers MLKL-dependent necroptosis in acidic TMEs, releasing DAMPs to activate STING signaling and synergize with ICIs against distant tumors (129–135). Similarly, Mn²^+^-enriched shikonin-loaded nanoparticles induce necroptosis in head and neck squamous cell carcinoma (HNSCC), disrupting mismatch repair to activate cGAS-STING-dependent IFN responses and enhance PD-1 blockade efficacy by promoting DC maturation and cytotoxic T cell infiltration (136–140). Beyond canonical RIPK1/RIPK3 pathways, the natural compound OSW-1 induces a RIP1/RIP3-independent necroptosis in colorectal cancer via p53-PUMA-CamKIIδ-MLKL signaling. This mechanism not only kills tumor cells but also sensitizes tumors to anti-PD-1 therapy by enhancing immunogenic antigen release (141). In postoperative non-small cell lung cancer (NSCLC), a photothermal hydrogel induces necroptosis in residual cancer cells, elevating DAMPs to polarize M1 macrophages and activate CD8^+^ T cell immunity, thereby preventing recurrence and infection (142). These advances underscore necroptosis as a versatile tool to reprogram immunosuppressive microenvironments and potentiate ICB through targeted induction strategies. Molecular orchestrations governing of necroptosis was shown in **Figure 2B**.

### 2.3 Overview of pyroptosis

Pyroptosis is an inflammatory RCD pathway characterized by GSDM family pore formation, which releases proinflammatory cytokines and induces cell lysis (63, 143, 144). Caspase-1, activated by canonical inflammasomes, cleaves Gasdermin D (GSDMD) to generate N-terminal fragments that oligomerize and perforate membranes (145–147). Noncanonical pathways involve caspase-4/5/11 or caspase-11 directly cleaving GSDMD upon cytosolic LPS detection (148–150). Apoptotic caspases can also trigger pyroptosis by cleaving GSDME, converting immunologically silent apoptosis into inflammatory death (151–153). In cancer, pyroptosis exhibits dual roles: GSDMD overexpression in NSCLC correlates with tumor aggressiveness (154–156), while NLRP3 activation in HCC suppresses metastasis (157–159). Therapeutic strategies leverage pyroptosis for antitumor immunity, inducing immunogenic death (72, 160). Additionally, GSDMB cleavage by granzyme in natural killer (NK) cells enhances tumor clearance, underscoring its potential in immunotherapy (161).

Pyroptosis has emerged as a potent strategy to remodel immunosuppressive TMEs and enhance ICB efficacy. In triple-negative breast cancer (TNBC), the ER stress sensor IRE1α suppresses taxane-induced immunogenicity by degrading double-stranded RNA (dsRNA) via regulated IRE1-dependent decay (RIDD), thereby inhibiting NLRP3 inflammasome-dependent pyroptosis. Pharmacological inhibition of IRE1α unleashes ZBP1-mediated recognition of dsRNA, activating NLRP3-GSDMD pyroptosis and converting PD-L1-negative tumors into immunogenic niches responsive to ICB (162). Similarly, in HCC, selective HDAC1/2/3 inhibition synergizes with ICB to enhance chromatin accessibility of IFNγ-responsive genes, promoting STAT1-driven pyroptosis via GSDME cleavage and amplifying cytotoxic T cell infiltration (157). Nanotechnology further expands pyroptosis applications: a tumor-specific nanoparticle undergoes charge reversal and nanofiber formation within lysosomes, disrupting lysosomal integrity to trigger GSDMD-mediated pyroptosis, which reverses immunosuppression and potentiates PD-L1 blockade in aggressive breast and pancreatic tumors (163). In gastric cancer (GC), transcriptional activation of GSDMD by HIC1 induces pyroptosis, releasing inflammatory cytokines to recruit CD8^+^ T cells, while combinatorial HIC1 overexpression with PD-L1 antibodies synergistically suppresses tumor growth (164). Natural compounds also harness pyroptosis: the Huangqin Houpo-derived combination induces GSDME-dependent pyroptosis, sensitizing colorectal cancer to anti-PD-1 therapy (165). Additionally, microwave-responsive AlEu-MOFs amplify NLR Family Pyrin Domain Containing 3 (NLRP3) inflammasome activation through HSP90 upregulation and ROS generation, achieving targeted pyroptosis to inhibit primary and metastatic breast tumors (166). These findings collectively underscore pyroptosis as a multifaceted immunomodulator, bridging innate immune activation with adaptive antitumor responses across diverse malignancies. Molecular orchestrations governing of pyroptosis was shown in **Figure 2C**.

### 2.4 Cross-talk among ferroptosis, necroptosis, and pyroptosis

The interplay between RCD pathways—necroptosis, pyroptosis, ferroptosis, and cuproptosis—synergistically amplifies ICD and reshapes tumor-immune dynamics (167–169). For instance, ZBP1 activation by viral RNA simultaneously triggers necroptosis via RIPK3-MLKL signaling (170) and pyroptosis through NLRP3 inflammasome activation (171), with MLKL pores releasing potassium to further enhance NLRP3-driven IL-1β secretion (172, 173). Similarly, copper ionophores like elesclomol disrupt mitochondrial copper homeostasis to induce cuproptosis while depleting GSH and downregulating Solute Carrier Family 7 Member 11 (SLC7A11), sensitizing cells to ferroptosis (174). Metabolic crosstalk further links these pathways: ACSL4-driven LPO in ferroptosis alters membrane composition to suppress pyroptosis, whereas necroptosis-induced DAMPs prime DCs to recognize pyroptosis-derived antigens. Therapeutically, co-targeting RIPK1 and GPX4 (ferroptosis) synergizes with PD-1 blockade to overcome immunotherapy resistance, while pyroptosis-inducing nanoparticles recruit neutrophils to enhance ferroptosis inducers within the TME. These interactions underscore the complexity of RCD networks and their potential to redefine cancer treatment through multimodal immunomodulation.

### 2.5 Emerging RCD pathways in melanoma

Malignant melanoma remains a formidable clinical challenge due to its high metastatic potential and profound resistance to conventional therapies, which primarily induce apoptosis. This resistance has catalyzed a paradigm shift in cancer research, moving the focus towards non-apoptotic forms of regulated cell death (RCD). Among these, ferroptosis, necroptosis, and pyroptosis have emerged as critical mechanisms that not only execute tumor cell killing but also actively modulate the tumor microenvironment (TME). Understanding the intricate molecular cross-talk among these pathways is proving essential for developing next-generation therapeutic strategies to overcome melanoma’s notorious plasticity.

Melanoma cells are susceptible to several non-apoptotic death modalities, each governed by a distinct molecular machinery. Ferroptosis is an iron-dependent form of RCD characterized by the overwhelming accumulation of lipid reactive oxygen species (ROS). Its execution is centrally regulated by the inhibition of glutathione peroxidase 4 (GPX4), a key enzyme that neutralizes lipid peroxides. Inducing ferroptosis has shown significant promise in overcoming resistance to targeted therapies and immunotherapy in melanoma models. Necroptosis, a form of programmed necrosis, is orchestrated by a signaling complex involving receptor-interacting protein kinase 1 (RIPK1), RIPK3, and the terminal executioner protein, mixed lineage kinase domain-like pseudokinase (MLKL). Upon activation, MLKL oligomerizes and translocates to the plasma membrane, forming pores that lead to cell lysis and the release of damage-associated molecular patterns (DAMPs). While some melanoma subtypes exhibit low sensitivity to necroptosis due to the downregulation of key components its induction can provoke a potent anti-tumor immune response, synergizing effectively with checkpoint blockade therapies.

Pyroptosis is a highly inflammatory RCD pathway mediated by the gasdermin (GSDM) family of pore-forming proteins. It is typically triggered by inflammasome activation, which leads to the cleavage of GSDMs by inflammatory caspases (e.g., caspase-1). The resulting pores cause cell swelling and lysis, accompanied by the massive release of pro-inflammatory cytokines such as IL-1β and IL-18, thereby robustly reshaping the TME. These RCD pathways do not operate in isolation but are extensively interconnected, forming a complex and adaptable cell death network. Key proteins function as nodes that integrate signals and dictate the ultimate cellular fate. RIPK1, the canonical initiator of necroptosis, also plays a crucial role in activating the NLRP3 inflammasome, directly linking necroptotic signaling to caspase-1 activation and pyroptosis. Furthermore, cellular stress signals like ROS serve as a common currency connecting these pathways. The intense lipid ROS production during ferroptosis can amplify necroptotic or pyroptotic signaling while mitochondrial dysfunction is a shared feature across all three RCDs. Caspases also act as critical regulators; for instance, caspase-8 can cleave and inactivate RIPK1 and RIPK3, thereby inhibiting necroptosis and potentially shunting the cell towards an alternative fate.

The profound cross-talk among ferroptosis, necroptosis, and pyroptosis presents novel therapeutic opportunities. The focus is shifting from inducing a single RCD pathway to orchestrating a multi-pronged attack that leverages their synergistic interactions. A particularly promising strategy involves the simultaneous induction of multiple immunogenic cell death pathways to maximize the anti-tumor immune response. Recent preclinical studies from 2023 and 2024 have showcased innovative approaches, such as nano-MOFs (metal-organic frameworks) designed to co-induce ferroptosis and pyroptosis in melanoma. This dual induction amplifies ROS production, enhances DAMP release, and potently stimulates an immune response, significantly improving the efficacy of immunotherapy. As of 2025, developing therapies that precisely manipulate these interconnected RCD nodes represents a cutting-edge approach to dismantle the formidable defenses of malignant melanoma.

## 3 Targeting ferroptosis, pyroptosis and necroptosis for cancer immunotherapy in melanoma

### 3.1 Targeting ferroptosis for cancer immunotherapy in melanoma

**Figure 3:**
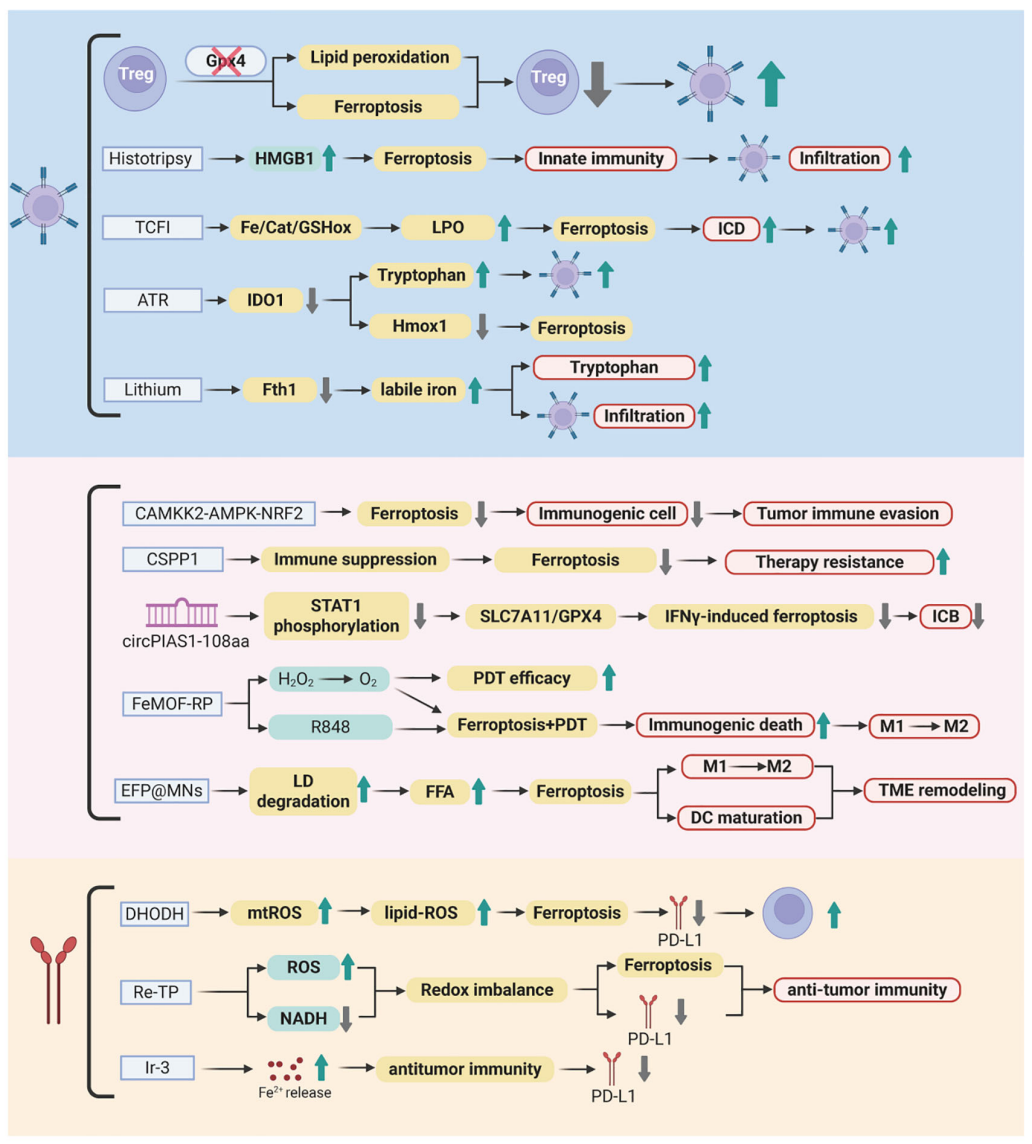
Ferroptosis-mediated immunotherapeutic landscape in melanoma. This figure illustrates the molecular interplay between ferroptosis induction and antitumor immunity. Key regulators including Gpx4, CAMKK2, and CSPP1 control lipid peroxidation, redox balance, and immune suppression within the tumor microenvironment. Pharmacological agents (B2, ART, lithium) and nanotechnology platforms (TCFI, FeMOF-RP, EFP@MNs) trigger iron-dependent cell death through ROS amplification, metabolic reprogramming, and immune checkpoint modulation. Synergistic integration with immune checkpoint inhibitors (anti-PD-1/PD-L1) enhances CD8+ T-cell activation, macrophage polarization, and systemic antitumor responses, overcoming therapeutic resistance. Created by Biorender.com.

Ferroptosis, driven by iron-dependent LPO, has emerged as a pivotal therapeutic target in melanoma, with its regulation intricately linked to both tumor cell vulnerability and immune modulation. This section synthesizes key molecular mechanisms and innovative strategies—from pharmacological agents to nanotechnology platforms—that harness ferroptosis to disrupt immunosuppression and enhance antitumor immunity. The synergy of ferroptosis inducers with ICB further highlights their potential to overcome therapeutic resistance. **Figure 3** and **Table 1** summarized these pathways and interventions, setting the stage for detailed exploration of ferroptosis-driven immunotherapy in melanoma.

**Table 1:** Molecular targets and therapeutic strategies for ferroptosis-driven immunotherapy in melanoma.

Target	Mechanism	Biological function	Therapeutic strategy	References
Gpx4	Gpx4 deletion in Treg cells induces lipid peroxidation and ferroptosis.	Suppresses Treg-mediated immunosuppression; enhances CD8+ T-cell anti-tumor immunity.	Genetic/pharmacological inhibition of Gpx4 in Treg cells.	(175)
CAMKK2	CAMKK2-AMPK-NRF2 axis suppresses ferroptosis.	Limits ferroptosis-driven immunogenic cell death and immune evasion.	CAMKK2 inhibitors combined with anti-PD-1.	(176)
CSPP1	CSPP1 regulates EMT, stromal remodeling, and immune responses.	Modulates tumor microenvironment (TME) to resist ferroptosis and immune checkpoint blockade.	Targeting CSPP1 to improve TME immunogenicity.	(177)
DHODH	Mitochondrial DHODH inhibitor B2 induces ROS and lipid peroxidation.	Triggers ferroptosis and downregulates PD-L1 to relieve immunosuppression.	B2 (DHODH inhibitor) as a mitochondrial-targeted ferroptosis inducer.	(178)
IDO1	Artemisinin (ART) inhibits IDO1 and Hmox1 transcription.	Induces ferroptosis and activates CD8+ T cells via tryptophan metabolism.	ART as an IDO1-targeting ferroptosis inducer.	(179)
Fth1	Lithium downregulates Fth1, increasing labile iron pools.	Enhances RSL3-induced ferroptosis and CD8+ T-cell infiltration.	Lithium + RSL3 combination therapy.	(180)
circPIAS1	circPIAS1-108aa inhibits STAT1 phosphorylation to suppress ferroptosis.	Limits IFNγ-induced immunogenic ferroptosis and ICB efficacy.	ASO-mediated circPIAS1 knockdown + anti-PD-1.	(181)
TCFI nanozyme	Triple enzymatic activity (Fenton, catalase, GSH oxidase) drives ferroptosis.	Induces immunogenic cell death and enhances CD8+ T-cell infiltration.	TCFI nanozyme + αPD-1.	(182)
FeMOF-RP nanozyme	Catalyzes H2O2 decomposition, releases oxygen, and delivers R848.	Combines ferroptosis with photodynamic therapy; polarizes M2→M1 macrophages.	FeMOF-RP nanozyme for dual ferroptosis and immune activation.	(183)
EFP@MNs microneedle	Degrades lipid droplets to boost lipid peroxidation and ferroptosis.	Reshapes TME via M1 macrophage polarization and DC maturation.	EFP@MNs + anti-PD-L1.	(184)
Histotripsy ultrasound	Triggers HMGB1 release and ferroptosis-associated immune activation.	Enhances CD8+ T-cell infiltration via innate immune stimulation.	Ultrasound + CTLA-4 blockade.	(185)
Re-TP rhenium complex	Generates ROS and disrupts redox balance via NADH oxidation.	Induces PD-L1 suppression and ferroptosis-driven anti-tumor immunity.	Re-TP + immunotherapy.	(186)
Ir-3 photoactivated prodrug	Releases Fe²^+^ to drive Fenton reaction and ICD.	Eliminates melanoma stem cells and prevents metastasis/recurrence.	Light-activated Ir-3 + immunotherapy.	(187)

#### 3.1.1 Molecular and immune mechanisms of ferroptosis regulation

Ferroptosis has garnered increasing interest as a potential therapeutic target in melanoma due to its complex regulation by both molecular and immune mechanisms. Notably, Gpx4 plays a pivotal role in protecting activated Treg cells from ferroptotic cell death, enabling these cells to maintain immune tolerance and suppress antitumor immunity. The genetic ablation of Gpx4 in Treg cells leads to the disruption of their immunosuppressive functions, enhancement of Th17 responses, and promotion of CD8^+^ T-cell-mediated tumor suppression. These findings underscore Gpx4 as a promising target for therapeutic intervention in melanoma (175).

Similarly, the kinase Calcium/Calmodulin-Dependent Protein Kinase Kinase 2 (CAMKK2), which functions through the AMPK-NRF2 axis, has been shown to negatively regulate ferroptosis in melanoma cells. Inhibition of CAMKK2 not only facilitates ferroptosis but also potentiates the efficacy of anti-PD-1 therapy, overcoming resistance to ICB (176). In addition to kinases, centrosome-associated protein CSPP1 emerges as a multifaceted regulator of ferroptosis and TME dynamics. Dysregulated CSPP1 expression is associated with EMT, stromal interactions, and immune checkpoint responsiveness, positioning CSPP1 as both a diagnostic biomarker and a potential therapeutic target (177).

Pharmacological strategies further illuminate the mechanistic pathways underlying ferroptosis regulation. The mitochondrial-targeted DHODH inhibitor B2 induces ferroptosis by generating ROS and promoting LPO, while simultaneously downregulating PD-L1 to alleviate immune suppression (178). Natural compounds such as artemisinin (ART) demonstrate dual functionality by directly targeting IDO1 to induce ferroptosis and enhancing CD8^+^ T-cell activity through modulation of tryptophan metabolism and PD-1 suppression (179). Lithium, a well-known pharmacological agent, sensitizes melanoma cells to ferroptosis by downregulating FTH, thereby increasing labile iron pools and synergizing with RSL3 to inhibit tumor growth and promote CD8^+^ T-cell infiltration (180). Additionally, circular RNA circPIAS1 acts as a barrier to ferroptosis-driven immunotherapy by encoding a peptide that suppresses STAT1 phosphorylation and reactivates the SLC7A11/GPX4 axis. Antisense oligonucleotide-mediated knockdown of circPIAS1 restores ferroptosis sensitivity and enhances the efficacy of PD-1 blockade, suggesting its potential as a therapeutic target (181).

#### 3.1.2 Therapeutic strategies for ferroptosis induction

Innovative therapeutic strategies have been developed to exploit ferroptosis induction in melanoma, offering new avenues for enhancing tumor immunotherapy. One promising approach involves the use of the TCFI nanozyme, which exhibits Fenton-like, catalase-like, and GSH oxidase-like activities. This nanozyme triggers LPO, alleviates hypoxia, and downregulates the GSH/GPX4 axis, synergizing with photodynamic therapy to enhance ROS generation and ICD. The TCFI nanozyme significantly increases CD8^+^ T-cell infiltration and interferon-γ (IFNγ) secretion, leading to potent suppression of both primary and metastatic tumors (182).

Another advanced strategy involves the FeMOF-RP nanosystem, which integrates photodynamic therapy with R848 delivery to catalyze intratumoral H_2_O_2_ decomposition. This process amplifies ferroptosis and promotes the polarization of immunosuppressive M2 macrophages toward an immunostimulatory M1 phenotype, thereby improving therapeutic outcomes (183). To address metabolic limitations within the TME, the EFP@MNs microneedle patch employs a lipolysis strategy to degrade lipid droplets and release free fatty acids (FFAs), fueling LPO. When combined with photothermal remodeling of the TME, this approach enhances DC maturation and synergizes with anti-PD-L1 therapy to achieve robust tumor ablation (184).

Non-chemical approaches have also demonstrated efficacy in triggering ferroptosis. Histotripsy, an ultrasound-based technique, induces ferroptosis-associated HMGB1 release and CD8^+^ T-cell infiltration. When combined with CTLA-4 blockade, histotripsy enhances antitumor effects (185). Metal complexes such as Re-TP exemplify the convergence of ferroptosis and immunotherapy. Re-TP, upon light activation, generates ROS, disrupts redox balance, and triggers PD-L1-related immune responses, leading to the eradication of both primary and metastatic tumors in murine models (186). Furthermore, the photoactivatable iridium complex Ir-3 releases Fe2^+^ to drive Fenton reactions and eliminate melanoma stem cells, thus preventing metastasis and recurrence (187).

#### 3.1.3 Combination with ICB

The integration of ferroptosis inducers with ICB has emerged as a promising strategy to enhance the efficacy of cancer immunotherapy. For instance, inhibition of CAMKK2 enhances ferroptosis and synergizes with anti-PD-1 therapy, effectively reversing immune evasion and overcoming therapeutic resistance (176). The TCFI nanozyme, when combined with αPD-1 antibodies, induces complete regression of primary tumors and suppresses distant metastases, highlighting the potential of nanotechnology-ICB combinations in melanoma therapy (182). Similarly, the EFP@MNs microneedle patch, when combined with anti-PD-L1 therapy, leverages lipolysis-driven ferroptosis and photothermal remodeling of the TME to enhance therapeutic outcomes (184).

Metal-based agents such as Re-TP further amplify the efficacy of ICB by inducing metabolic reprogramming, promoting ferroptosis and ICD while simultaneously suppressing PD-L1 expression (186). Collectively, these studies underscore the potential of ferroptosis-ICB combination therapies to achieve durable antitumor immunity and address the challenges of tumor heterogeneity and resistance mechanisms.

### 3.2 Targeting necroptosis for cancer immunotherapy in melanoma

**Figure 4:**
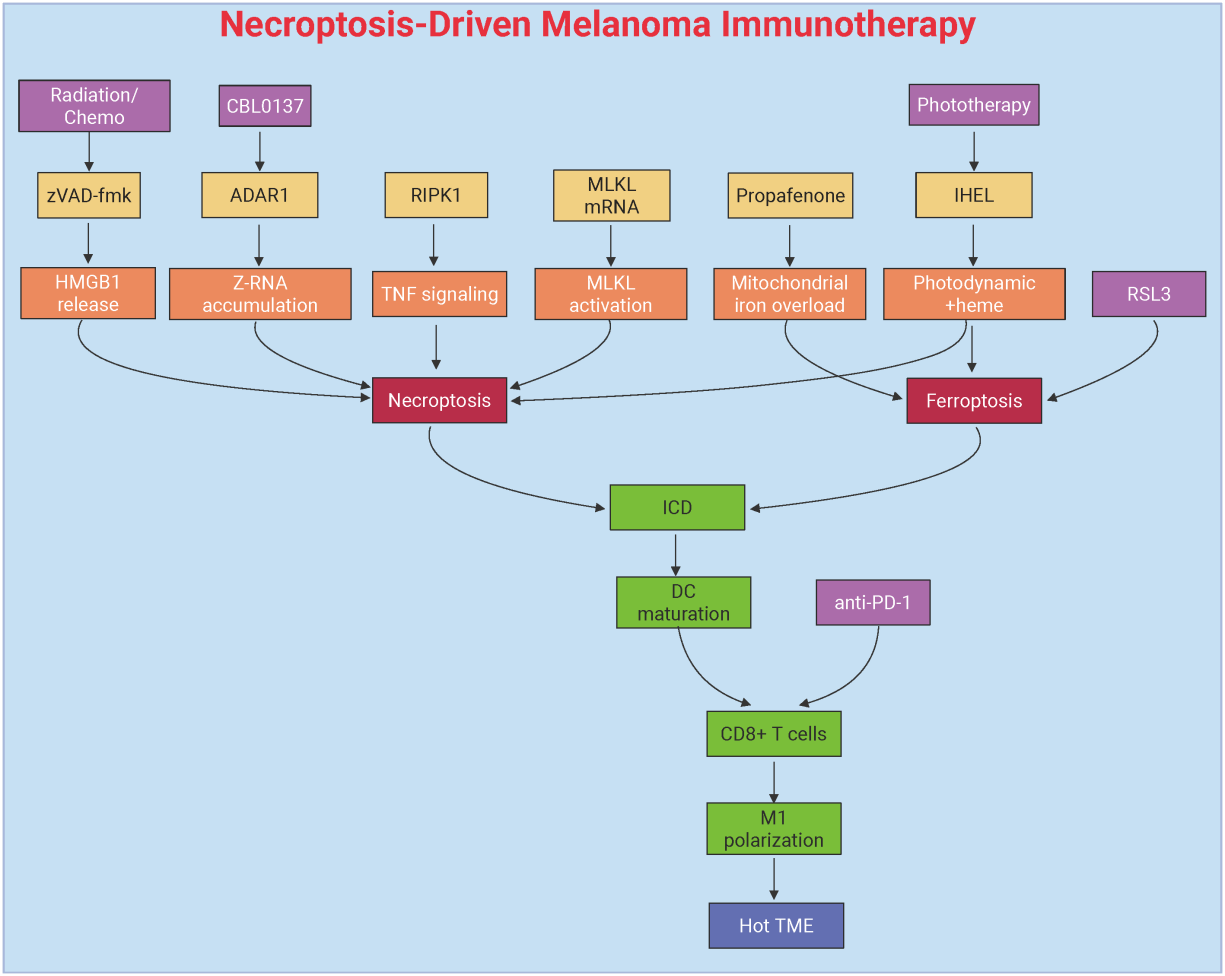
Necroptosis-mediated immunotherapeutic modulation in melanoma. This figure delineates necroptosis-driven antitumor immunity through DAMPs release and dendritic cell activation. Key interventions include pharmacological agents (propafenone) inducing JNK/JUN-mediated iron overload, mRNA-based MLKL delivery bypassing upstream signaling defects, and IHEL nanovesicles combining phototherapy with heme-induced necroptosis. Synergy with immune checkpoint blockade (anti-PD-1/PD-L1) amplifies T-cell infiltration, remodels the immunosuppressive tumor microenvironment, and suppresses metastasis through RIPK1-dependent immunogenic cell death and macrophage polarization. Created by Biorender.com.

Necroptosis, a lytic and ICD pathway, has emerged as a key driver of antitumor immunity in melanoma by releasing DAMPs and activating DC-mediated T cell responses. This section highlights molecular regulators and therapeutic strategies—from repurposed drugs to mRNA-based MLKL delivery and hybrid nanovesicles—that induce necroptosis to overcome immune evasion. Synergy with ICB further enhances T cell infiltration and suppresses metastasis. **Figure 4** and **Table 2** summarized these mechanisms and interventions, setting the stage for exploring necroptosis-centered immunotherapies in melanoma.

**Table 2:** Molecular targets and therapeutic strategies for necroptosis-driven immunotherapy in melanoma.

Target	Mechanism	Biological function	Therapeutic strategy	References
Caspases (pan-inhibition)	zVAD-fmk inhibits apoptosis, shifting cell death to necroptosis with HMGB1 release.	Enhances immunogenic cell death and activates DCs/CD8+ T cells via MyD88-dependent pathways.	zVAD-fmk combined with multimodal therapy (radiotherapy/chemotherapy/hyperthermia).	(188)
ADAR1-ZBP1 axis	ADAR1 deletion leads to Z-RNA accumulation, activating ZBP1-mediated necroptosis.	Overcomes ICB resistance by restoring immunogenic necroptosis.	CBL0137 (ZBP1 activator) to bypass ADAR1-mediated immunosuppression.	(137)
Propafenone	Activates JNK/JUN-HMOX1 axis, inducing mitochondrial iron overload and ROS.	Sensitizes melanoma to necroptosis and synergizes with immunotherapy.	Propafenone + necroptosis inducers (e.g., RSL3).	(189)
MLKL mRNA	Local delivery of MLKL-mRNA bypasses upstream necroptosis signaling defects.	Triggers tumor-specific T-cell responses via type I IFN and Batf3+ DCs.	MLKL-mRNA + immune checkpoint blockade (e.g., anti-PD-1/CTLA-4).	(190)
IHEL nanoparticles	Combines phototherapy (IR780) with heme-induced necroptosis and metabolic modulation.	Converts “immune-cold” to “immune-hot” TME via M1 macrophage polarization.	IHEL system for dual necroptosis induction and tumor microenvironment remodeling.	(191)
RIPK1	T-cell-derived TNF activates RIPK1-dependent necroptosis in target cells.	Enhances anti-PD1 efficacy by promoting immunogenic cell death.	TNF-RIPK1 axis modulation + anti-PD1.	(192)

#### 3.2.1 Molecular mechanisms of necroptosis-driven immune activation

Necroptosis has gained significant attention as a mechanism to stimulate antitumor immunity in melanoma. This process is characterized by the activation of several key immune pathways, making it a promising strategy for overcoming immune evasion in melanoma. One of the critical factors influencing necroptosis in melanoma is the pan-caspase inhibitor Z-Val-Ala-Asp(OMe)-FMK (zVAD-fmk). This compound enhances the immunogenicity of tumor cell death by shifting the cell death pathway from apoptosis to necroptosis, leading to increased release of DAMPs, such as HMGB1. These DAMPs, in turn, activate macrophages and DCs via MyD88-, nucleotide-, and T cell-dependent mechanisms. In murine melanoma models, the combination of zVAD-fmk with multimodal therapies has been shown to significantly reduce tumor growth, decrease regulatory T cell infiltration, and promote the recruitment of CD8^+^ T cells along with enhanced IFNγ expression. These effects indicate that necroptosis can remodel the immunosuppressive TME, paving the way for more effective cancer immunotherapy (188).

Another critical regulator of necroptosis-driven immune activation is the RNA-editing enzyme Adenosine Deaminase Acting on RNA 1 (ADAR1), which suppresses the accumulation of endogenous Z-RNA. ADAR1 deficiency, by allowing the accumulation of Z-RNA, triggers necroptosis through the Z-RNA sensor Z-DNA Binding Protein 1 (ZBP1), effectively bypassing resistance mechanisms to ICB. Pharmacological activation of ZBP1 using the small molecule CBL0137 has been shown to restore ICB responsiveness in melanoma. This is achieved by inducing the expression of IFN-stimulated genes (ISGs) and promoting necroptotic cell death, highlighting ADAR1 as a potential therapeutic target to overcome immune evasion in melanoma (137).

#### 3.2.2 Therapeutic strategies to induce necroptosis

Targeted induction of necroptosis has shown significant promise in overcoming therapy resistance in melanoma. One such approach involves the use of the antiarrhythmic drug propafenone, which sensitizes melanoma cells to necroptosis by activating the JNK/JUN signaling pathway, upregulating mitochondrial heme oxygenase 1 (HMOX1), and inducing iron overload and ROS accumulation. Propafenone has been shown to synergize with necroptosis inducers, such as RSL3, resulting in near-complete tumor regression in xenograft models. Additionally, propafenone enhances ICB efficacy by promoting tumor cell necroptosis and increasing T cell infiltration. Clinically, melanoma patients exhibiting elevated necroptosis-related signatures, such as JUN and HMOX1 expression, show improved responses to ICB therapy and prolonged progression-free survival (189).

mRNA-based therapies, such as intratumoral delivery of MLKL-encoding mRNA, represent another promising strategy for inducing necroptosis. This approach bypasses upstream signaling defects in necroptosis and directly triggers robust antitumor immunity. MLKL-mRNA therapy induces tumor neoantigen-specific T cell responses, which are dependent on type I IFN signaling and Batf3^+^ DCs. When combined with ICB, MLKL-mRNA therapy effectively suppresses both primary and metastatic tumors, highlighting its potential as a valuable adjunct to existing immunotherapeutic strategies (190).

Hybrid exosome/liposome nanovesicles are another innovative strategy that integrates multiple therapeutic modalities, combining phototherapy with heme-induced necroptosis to overcome apoptosis resistance. IHEL nanovesicles not only induce necroptosis but also remodel the TME by alleviating hypoxia, inhibiting glycolysis, and polarizing macrophages towards an immunostimulatory M1 phenotype. These effects contribute to enhanced antitumor immunity, making IHEL a versatile platform for necroptosis-centered therapies (191).

#### 3.2.3 Synergy with ICB

The combination of necroptosis induction with ICB represents a promising therapeutic approach for melanoma. T cell-derived TNFα has been shown to activate RIPK1-dependent necroptosis in target cells, a critical mechanism for accelerating allograft rejection and amplifying the antitumor effects of anti-PD1 therapy. In melanoma models, RIPK1-dependent necroptosis works synergistically with ICB by promoting ICD and enhancing T cell activation, thus amplifying the efficacy of ICIs (192).

MLKL-mRNA therapy further exemplifies this synergy, as its combination with ICB leads to enhanced CD8^+^ T cell-mediated tumor clearance and the establishment of durable immune memory. This approach leverages both direct tumor cell killing and the priming of antitumor immunity to improve therapeutic outcomes (190). Similarly, propafenone-induced necroptosis has been shown to reshape the TME to favor ICB responsiveness. By linking necroptotic cell death with adaptive immune activation, propafenone enhances the antitumor effects of ICB therapy (189).

These findings collectively underscore the dual role of necroptosis in both directly eliminating tumor cells and indirectly enhancing antitumor immunity. By modulating the TME and promoting immune activation, necroptosis is poised to be a cornerstone of next-generation combination therapies that aim to overcome resistance and improve the durability of cancer treatments.

### 3.3 Targeting pyroptosis for cancer immunotherapy in melanoma

**Figure 5:**
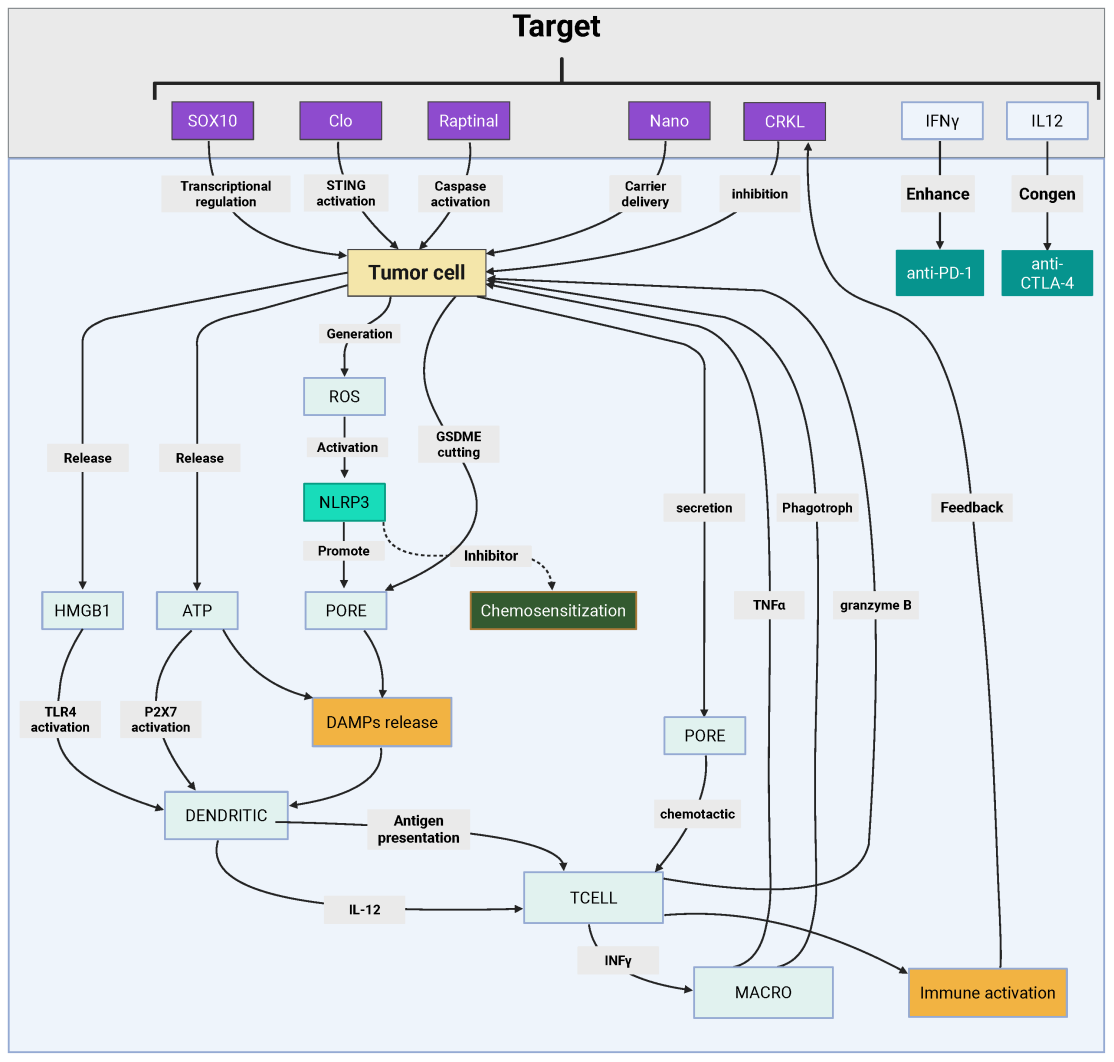
Pyroptosis-centric immunotherapeutic integration in melanoma. This figure depicts pyroptosis-mediated antitumor immunity through gasdermin pore-driven DAMPs release and adaptive immune activation. Key strategies include pharmacological activators (clofarabine, raptinal) modulating STING-NF-κB/GSDME pathways, nanotechnology platforms (R@AZOH, Nano-CD) coupling CRISPR-based GSDME induction with innate immunity priming, and biomimetic nanoparticles enhancing chemotherapeutic pyroptosis. Synergy with BRAF/MEK inhibitors and immune checkpoint blockade amplifies CD8+ T-cell infiltration via HMGB1-mediated immunogenicity while remodeling immunosuppressive niches. Prognostic pyroptosis scoring (PScore) stratifies patients by immune microenvironment features and therapeutic responsiveness, guiding precision immunotherapy design. Created by Biorender.com.

Pyroptosis, an inflammatory and ICD pathway driven by GSDM family pore formation, has emerged as a pivotal strategy to reignite antitumor immunity in melanoma by releasing DAMPs and activating adaptive immune responses. This section outlines key molecular regulators and therapeutic innovations—from pharmacological agents to CRISPR-based platforms and biomimetic nanoparticles—that leverage pyroptosis to counter immune evasion. Synergy with ICB and targeted therapies further amplifies CD8^+^ T cell infiltration and remodels the immunosuppressive TME. Prognostic models like the pyroptosis score (PScore) provide insights into patient stratification and ICB responsiveness. **Figure 5** and **Table 3** summarize these pathways and interventions, setting the stage for detailed exploration of pyroptosis-driven immunotherapies in melanoma.

**Table 3:** Molecular targets and therapeutic strategies for pyroptosis-driven immunotherapy in melanoma.

Target	Mechanism	Biological function	Therapeutic strategy	References
CRKL	CRKL inhibition activates pyroptosis-related pathways.	Predicts anti-PD1 response; correlates with immune activation and poor prognosis.	CRKL-targeted therapies to enhance pyroptosis.	(193)
SOX10	SOX10 deletion sensitizes melanoma to cytokine-induced pyroptosis.	Enhances CD8+ T-cell-mediated killing via caspase-dependent pyroptosis.	Targeting SOX10 to potentiate T-cell immunity.	(194)
Clofarabine (Clo)	Activates non-canonical STING-NF-κB pathway, upregulating HLA/BAX and chemokines.	Induces pyroptosis and immunogenic cell death (ICD) in melanoma and lung cancer.	Repurposing Clo for STING-NF-κB-driven pyroptosis.	(195)
ACSL4-mediated lipid rafts	Lipid raft formation suppresses pyroptosis by sequestering immunogenic pathways.	Reduces sensitivity to platinum-based ICD.	Disrupting lipid rafts (e.g., cholesterol depletion) to restore pyroptosis.	(196)
Raptinal	Caspase-3/GSDME-dependent pyroptosis in BRAF-mutant melanoma.	Overcomes therapy resistance by inducing inflammatory cell death.	Raptinal as a pyroptosis inducer for BRAF/MEKi-resistant tumors.	(197)
R@AZOH nanozyme	Zinc-induced mitochondrial dysfunction triggers pyroptosis and DAMPs release.	Synergizes phototherapy with TLR7/8 activation to remodel TME.	R@AZOH for dual pyroptosis induction and DC maturation.	(198)
Nano-CD platform	CRISPR/dCas9-driven GSDME expression + cisplatin-induced caspase-3 activation.	Reverses immunosuppressive TME via pyroptosis-ICD synergy.	Nano-CD + immune checkpoint blockade (ICB).	(199)
ZIF-8 nanoparticles	DCT-enhanced GSDME expression enables OXA-induced pyroptosis.	Creates pro-inflammatory TME via pyroptosis and R837-mediated immune activation.	Biomimetic nanoparticles for chemotherapy-pyroptosis combination.	(200)
CSCCPT/SNAP nanodrug	ROS/NO synergism triggers pyroptosis and sustains antitumor immunity.	Amplifies pyroptosis via reactive nitrogen species (RNS).	Supramolecular nanomedicine for self-reinforcing pyroptosis.	(201)
Ir(III)-C3N5 nanocomposite	Piezocatalytic ROS generation induces lysosomal rupture and pyroptosis.	Reverses hypoxic TME and enhances antigen presentation.	Oxygen-self-sufficient nanoplatform for sonodynamic immunotherapy.	(202)
4S5NG-PE24 conjugate	Integrin α6-targeted delivery of PE24 induces pyroptosis.	Promotes antitumor immunity via pyroptosis-dependent T-cell activation.	Peptide-drug conjugates for precision pyroptosis induction.	(203)
BRAFi + MEKi	Immunogenic pyroptosis via GSDME cleavage and HMGB1 release.	Enhances T-cell infiltration and overcomes targeted therapy resistance.	BRAFi + MEKi combined with pyroptosis-inducing chemotherapy.	(204)
PDPK1	PDPK1 inhibition synergizes with MEKi to induce pyroptosis.	Increases intratumoral CD8+ T cells in NRAS-mutant melanoma.	PDPK1 inhibitors + MEKi for immunostimulatory pyroptosis.	(205)
CDNP nanogel	Dabrafenib + celecoxib co-delivery induces pyroptosis and TME remodeling.	Synergizes BRAF/COX2 inhibition with pyroptosis-driven immune activation.	CDNP + anti-PD1 for enhanced melanoma control.	(206)
MS-275 + V-9302	Epigenetic-metabolic co-targeting triggers ROS-dependent pyroptosis.	Converts “cold” to “hot” TME in uveal melanoma.	ROS-sensitive nanoparticles + anti-PD1.	(207)
H101 adenovirus	Caspase-1/GSDMD-mediated endothelial pyroptosis.	Reduces M2 macrophages and enhances CD8+ T-cell infiltration.	H101 + anti-PD-L1 for synergistic tumor suppression.	(208)
pH-sensitive nanoplatform	ER stress-pyroptosis-ferroptosis crosstalk via metabolic modulation.	Polarizes TAMs to M1 phenotype and induces cytotoxic T-cell responses.	Multimodal nanotherapy + ICB for durable antitumor immunity.	(209)
PRGs (GZMA+/GSDMB+)	Reduced pyroptosis capacity in CD8+ T/NK cells correlates with melanoma progression.	Prognostic risk model links PRGs to immune evasion.	PRG-based nomogram for predicting survival and therapy response.	(210)
Pyroptosis Score (PScore)	High PScore correlates with immune-rich TME and better ICB response.	Predictive biomarker for metastatic melanoma survival and immunotherapy efficacy.	PScore-guided stratification to optimize ICB outcomes.	(211)

#### 3.3.1 Molecular mechanisms and regulatory pathways

Pyroptosis, a lytic and inflammatory form of PCD, is intricately regulated by molecular pathways that significantly intersect with tumor immunity in melanoma. One of the key regulators identified is the proto-oncogene C-reaK-tyrosine kinAse like (CRKL), which plays a critical role in melanoma progression. Overexpression of CRKL has been shown to correlate with poor prognosis and reduced responsiveness to anti-PD1 therapy, positioning it as both a prognostic biomarker and a potential therapeutic target. Suppression of CRKL activates pyroptosis-related pathways, highlighting its pivotal role in melanoma immune evasion (193).

Similarly, the transcription factor (Sex Determining Region Y)-box 10 (SOX10) modulates melanoma cell susceptibility to T cell-mediated killing. SOX10 deficiency sensitizes melanoma cells to pyroptosis induced by TNFα and IFNγ, triggering caspase-dependent mechanisms that enhance ICD. This process reshapes the melanoma tumor-immune interaction, promoting the activation of antitumor immunity (194).

Pharmacological agents such as clofarabine (Clo) have been identified as key enhancers of the link between apoptosis and pyroptosis. Clo activates the non-canonical STING-NF-κB pathway, inducing pyroptosis and upregulating HLA molecules and chemokines such as CCL5 and CXCL10. These changes amplify the recruitment and activation of CD8^+^ T cells, thereby enhancing antitumor immunity (195). Conversely, the formation of lipid rafts through ACSL4 activity has been shown to suppress platinum-induced pyroptosis. Notably, cholesterol depletion can restore ICD, enhancing the efficacy of chemotherapy (196). Collectively, these findings underscore the complex interplay between pyroptosis regulators and immune evasion mechanisms, highlighting potential therapeutic opportunities.

#### 3.3.2 Therapeutic strategies for pyroptosis induction

A variety of innovative therapeutic platforms have been developed to exploit pyroptosis in melanoma treatment. One such approach involves the use of the caspase-3 activator raptinal, which induces pyroptosis in BRAF-mutant melanoma through GSDME cleavage. This mechanism overcomes resistance to BRAF/MEK inhibitors (BRAFi/MEKi) and delays tumor growth, suggesting its potential as a therapeutic strategy in resistant melanoma (197).

Nanotechnology-based strategies, such as R848-loaded AlZn hydroxide (R@AZOH), combine pyroptosis induction with innate immune activation. R@AZOH triggers zinc-dependent mitochondrial dysfunction, promoting the release of DAMPs and supporting DC maturation. This promotes sustained adaptive immunity, further strengthening the antitumor response (198).

CRISPR-based strategies also offer a promising approach to enhancing pyroptosis in melanoma. The Nano-CD platform exploits endogenous GSDME expression via tumor-supplied CRISPR/dCas9 systems. When combined with cisplatin-induced caspase-3 activation, Nano-CD induces robust pyroptosis, reverses the immunosuppressive TME, and synergizes with ICB (199).

Additionally, biomimetic nanoparticles, such as ZIF-8 coated with cancer cell membranes, enhance oxaliplatin delivery and GSDME-mediated pyroptosis by inducing GSDME upregulation through DNA methyltransferase inhibition (200). Advanced nanomedicines like CSCCPT/SNAP, which generate reactive nitrogen species (RNS), also amplify pyroptosis. Furthermore, iridium (III)-C3N5 nanocomposites induce lysosomal rupture and pyroptosis via sonodynamic therapy, effectively overcoming hypoxia and enhancing the therapeutic response (201, 202).

Targeted delivery systems, such as the integrin α6-specific cell-penetrating peptide 4S5NG, enable precise induction of pyroptosis in melanoma cells while sparing normal tissues, thereby increasing the safety and efficacy of treatment (203). These diverse strategies highlight the versatility and potential of pyroptosis-centered approaches in melanoma immunotherapy.

#### 3.3.3 Combination therapies with immunotherapy/targeted agents

The combination of pyroptosis inducers with targeted therapies or ICB has shown transformative potential in melanoma treatment. BRAFi/MEKi induce pyroptosis via GSDME cleavage and HMGB1 release, activating CD8^+^ T cell-dependent antitumor immunity. However, resistance to BRAFi/MEKi therapy is often associated with diminished pyroptosis markers, suggesting that enhancing pyroptosis could overcome therapeutic escape and improve treatment outcomes (204).

Similarly, PDPK1 inhibition has been shown to synergize with MEK inhibitors in NRAS-mutant melanoma, enhancing pyroptosis and promoting CD8^+^ T cell infiltration in immunocompetent models (205). Nanotherapeutic platforms, such as the BRAFi/COX2i-loaded nanogel Cyclic di-nucleotide phosphodiesterase (CDNP), induce pyroptosis and remodel the TME, leading to superior tumor control when combined with anti-PD1 therapy (206).

ROS-sensitive nanoparticles, co-delivering HDAC and glutamine metabolism inhibitors, have also been shown to convert “immunologically cold” tumors into “hot” tumors by triggering pyroptosis and enhancing PD-1 blockade efficacy (207). Additionally, viral therapies such as the oncolytic adenovirus H101 induce endothelial pyroptosis via caspase-1/GSDMD activation, synergizing with anti-PD-L1 therapy to suppress tumor growth (208). Pioneering pH-sensitive nanoplatforms demonstrate multimodal induction of pyroptosis and ferroptosis, polarizing tumor-associated macrophages (TAMs) and enhancing ICB responses (209). These studies illustrate the potential of combining pyroptosis with immune modulation to overcome therapeutic resistance and improve treatment outcomes in melanoma.

#### 3.3.4 Prognostic models and TME insights

Single-cell transcriptomic analyses have provided valuable insights into the dysregulated expression of pyroptosis-related genes (PRGs) in melanoma, particularly within CD8^+^ T and NK cells. The reduced presence of GZMA^+^ CD8^+^ T cells and GSDMB^+^ NK cells in tumors is associated with a diminished pyroptotic capacity, indicating that immune cell pyroptosis plays a role in melanoma progression (210).

The PScore, a computational model integrating PRG expression, has emerged as a potential tool for predicting patient survival and response to ICB therapy in metastatic melanoma. Tumors with a high PScore are characterized by an immune-enriched TME and show better responses to ICB therapy, while low PScore tumors are associated with fibrotic or immune-exhausted microenvironments. Notably, monocytes exhibit the highest PScore, contrasting with malignant cells and fibroblasts, which are resistant to pyroptosis (211). These prognostic models provide a framework for stratifying patients and tailoring pyroptosis-centered immunotherapies, offering a more personalized approach to melanoma treatment.

### 3.4 Targeting cross-mechanism interactions for immunomodulation in melanoma

The interplay of RCD pathways and metabolic reprogramming offers a multidimensional strategy to enhance antitumor immunity in melanoma. This section explores how synergistic activation of ICD, single-cell-guided insights into tumor heterogeneity, and metabolic interventions reshape the immunosuppressive microenvironment. By integrating these cross-mechanism approaches, novel therapeutic strategies aim to overcome resistance and amplify immunotherapy efficacy. **Table 4** summarized these interactions, setting the stage for detailed discussion in the following sections to highlight the synergies and challenges of co-targeting multiple PCD pathways.

**Table 4:** Molecular targets and therapeutic strategies for cross-mechanism immunomodulation in melanoma.

Target	Mechanism	Biological function	Therapeutic strategy	References
TPL@TFBF nanozyme	Synergistic ferroptosis/pyroptosis via Fenton reaction-driven ROS accumulation.	Releases DAMPs to activate DCs and cytotoxic T cells, suppressing tumor growth and metastasis.	TPL@TFBF + immune checkpoint blockade.	(212)
ACSL4-mediated lipid rafts	Lipid raft formation suppresses ferroptosis/pyroptosis by sequestering immunogenic pathways.	Reduces sensitivity to platinum-based ICD.	Cholesterol depletion to disrupt lipid rafts.	(196)
C0 SOD3+ melanoma subpopulation	Oxidative pathway-enriched metastatic subpopulation linked to multiple PCD forms (apoptosis, autophagy, ferroptosis, pyroptosis).	Correlates with immune evasion and poor prognosis.	Prognostic model integrating PCD-related genes.	(213)
Decitabine	Hypomethylating agent reverses immunosuppression by restoring IFN/ISG expression.	Restores CD8+ T-cell function and reduces myeloid-derived suppressor cells (MDSCs).	Low-dose decitabine to reprogram immunosuppressive TME.	(214)
Lipid starvation scaffold	Inhibits fatty acid uptake via FATP/TGF-β blockade, polarizing neutrophils to N1 phenotype.	Triggers pyroptosis, recruits M1 macrophages, and amplifies antitumor immunity.	ECM-mimetic scaffold delivering FATP/TGF-β inhibitors.	(215)

#### 3.4.1 The diverse landscape of regulated cell death in melanoma

Melanoma is characterized by its profound resistance to conventional cell death mechanisms, a hallmark of its aggressive nature. While apoptosis, or Type I programmed cell death (PCD), is the most studied pathway, melanoma cells frequently upregulate anti-apoptotic machinery, rendering them insensitive to therapies designed to trigger it. This inherent resistance has driven researchers to explore and exploit non-apoptotic forms of regulated cell death (RCD) as alternative therapeutic avenues. These pathways not only offer direct tumor-killing capabilities but can also be highly immunogenic, fundamentally altering the dialogue between the dying cancer cell and the host immune system (212).

Several non-apoptotic RCD pathways have emerged as critical players in melanoma biology and therapy.

##### 3.4.1.1 Necroptosis

A caspase-independent form of programmed necrosis orchestrated by the RIPK1/RIPK3/MLKL signaling axis. Unlike the immunologically silent process of apoptosis, necroptosis results in cell lysis and the release of damage-associated molecular patterns (DAMPs), which can powerfully stimulate an anti-tumor immune response. Inducing necroptosis has been shown to synergize with immunotherapy to enhance anti-melanoma immunity. For instance, direct intratumoral delivery of MLKL mRNA can inhibit melanoma growth and boost the efficacy of immune checkpoint inhibitors (ICIs).

##### 3.4.1.2 Ferroptosis

An iron-dependent form of cell death characterized by the overwhelming accumulation of lipid peroxides. This pathway is intricately linked to cellular metabolism, particularly lipid and iron homeostasis, making it a unique target in metabolically rewired cancer cells. Ferroptosis has demonstrated significant potential in overcoming therapy resistance in melanoma, including resistance to BRAF inhibitors and immunotherapy. Combining ferroptosis inducers with ICIs like anti-PD-1/PD-L1 antibodies has shown synergistic anti-tumor effects.

##### 3.4.1.3 Pyroptosis

A highly inflammatory form of programmed cell death mediated by the gasdermin protein family, leading to cell swelling, lysis, and the release of pro-inflammatory cytokines. This robust inflammatory response can elicit a strong adaptive immune response, promoting the infiltration of immune cells into the tumor and correlating with a favorable prognosis in melanoma patients.

##### 3.4.1.4 PANoptosis

A more recently described inflammatory cell death pathway that integrates key features of pyroptosis, apoptosis, and necroptosis. This pathway can be activated by the sensor protein ZBP1 and has been shown to repress melanoma growth and improve responsiveness to immune checkpoint blockade.

The ability to trigger these diverse death modalities provides a strategic advantage, allowing for tailored approaches that bypass melanoma’s specific anti-apoptotic defenses (196).

#### 3.4.2 Immunogenic cell death: transforming tumors into endogenous vaccines

The ultimate goal of many modern cancer therapies is not just to kill tumor cells but to do so in a way that stimulates a lasting, systemic anti-tumor immune response. This is the central concept of Immunogenic Cell Death (ICD). ICD is a form of RCD where dying cancer cells release a specific set of DAMPs that act as potent adjuvants, effectively turning the tumor into an *in-situ* vaccine (213).

The canonical process of ICD involves several key steps: (1) Exposure of “Eat-Me” Signals: Dying cells expose calreticulin (CRT) on their surface, signaling phagocytic cells like dendritic cells (DCs) to engulf them. (2)Release of “Find-Me” Signals: The release of ATP attracts antigen-presenting cells (APCs) to the tumor site. (3)Activation of Immune Sensors: The release of High Mobility Group Box 1 (HMGB1) protein binds to Toll-like receptor 4 (TLR4) on DCs, promoting their maturation and antigen presentation capabilities.

This cascade of events transforms an immunosuppressive tumor microenvironment (TME) into an immunostimulatory one. Mature DCs migrate to draining lymph nodes, where they present tumor antigens to naive T cells, priming a robust, tumor-specific cytotoxic T lymphocyte (CTL) response. These CTLs then infiltrate the tumor, recognize and eliminate remaining cancer cells, and can form a long-term immunological memory.

Therapeutic strategies including certain chemotherapies, radiotherapy, and photodynamic therapy are known ICD inducers. The true power of ICD, however, lies in its synergy with immunotherapy. By generating a fresh wave of tumor-specific T cells, ICD can sensitize “cold” tumors (lacking immune infiltrate) to ICIs like anti-PD-1 and anti-CTLA-4 antibodies, which work by “releasing the brakes” on already-present T cells. This combination can overcome primary and acquired resistance to immunotherapy, a major clinical challenge in melanoma treatment (214).

#### 3.4.3 Metabolic reprogramming: the engine of malignancy and an immunosuppressive force

Disordered metabolism is a core hallmark of cancer, and melanoma is a prime example of a tumor that extensively rewires its metabolic pathways to support rapid proliferation, survival, and metastasis. This metabolic reprogramming, often driven by oncogenic mutations such as in the BRAF gene, is not just a cell-intrinsic process; it profoundly shapes the TME and orchestrates immune evasion.

Melanoma cells exhibit high rates of aerobic glycolysis (the Warburg effect), diverting glucose away from oxidative phosphorylation (OXPHOS) to produce lactate and biosynthetic precursors. This metabolic shift creates a TME that is acidic and nutrient-deprived (e.g., low in glucose and tryptophan), which directly impairs the function of effector immune cells like T cells and NK cells, while favoring the function of immunosuppressive cells like regulatory T cells (Tregs) and myeloid-derived suppressor cells (MDSCs). Beyond glycolysis, melanoma cells also exhibit altered lipid, amino acid, and glutathione metabolism, all of which contribute to both tumor growth and the suppression of antitumor immunity.

This deep connection between metabolism and immunity presents a therapeutic vulnerability. Targeting key metabolic enzymes or pathways is emerging as a promising strategy to not only directly inhibit tumor growth but also to normalize the TME, thereby restoring the efficacy of immune cells and enhancing the effects of immunotherapy (215).

## 4 Discussion and future direction

The therapeutic landscape for advanced melanoma has been fundamentally reshaped over the past decade by the advent of immunotherapy, particularly immune checkpoint inhibitors (ICIs). Despite these transformative successes, a significant portion of patients exhibit primary or acquired resistance, creating an urgent need for novel strategies that can overcome these limitations (62, 63, 72). A promising new frontier in oncology involves the deliberate induction of specific forms of regulated cell death (RCD) to not only eliminate tumor cells directly but also to potently stimulate a robust and durable anti-tumor immune response.

This report focuses on the emerging paradigm of simultaneously targeting three distinct, non-apoptotic RCD pathways—ferroptosis, pyroptosis, and necroptosis—as a comprehensive strategy to enhance cancer immunotherapy in melanoma. These pathways, once studied in isolation, are now understood to be interconnected components of a broader “cell death network” that can be manipulated to convert an immunologically “cold” tumor microenvironment (TME) into a “hot” one, thereby sensitizing melanoma to immune-mediated destruction (216, 217). We will delve into the mechanistic underpinnings of each pathway, explore the rationale for their combined targeting, analyze the technological advancements facilitating this research, and critically evaluate the significant challenges and future directions on the path to clinical translation (53, 218–221).

Recent studies have demonstrated the potential of combining different forms of PCD, such as ferroptosis, pyroptosis, and necroptosis, to enhance ICD and overcome resistance to conventional therapies. By targeting these pathways simultaneously, researchers have designed multifunctional nanoparticles, small-molecule combinations, and metabolic interventions that not only induce tumor cell death but also reshape the immunosuppressive TME. For example, combining ferroptosis inducers with agents that activate pyroptosis has been shown to release DAMPs and cytokines, which in turn recruit DCs and CTLs, thereby enhancing systemic antitumor immunity (222, 223). Similarly, metabolic interventions that target lipid or iron homeostasis can deplete essential tumor resources while reprogramming immune cells toward pro-inflammatory phenotypes, presenting a promising avenue to augment therapeutic efficacy.

Additionally, cutting-edge technologies such as single-cell omics and spatial transcriptomics have offered an in-depth understanding of melanoma’s heterogeneity and its immune landscape (32, 46, 224–226). These approaches have revealed rare subpopulations of therapy-resistant melanoma cells and highlighted the dynamic shifts in immune cell states during treatment. This high-resolution analysis has facilitated the design of precision therapies aimed at vulnerabilities in both malignant and stromal compartments. Moreover, the repurposing of FDA-approved drugs, such as decitabine and propafenone, to modulate epigenetic and metabolic pathways underscores the potential to translate mechanistic insights into clinically actionable therapies (189, 227, 228).

Despite these promising developments, there are several barriers that need to be addressed before preclinical findings can be successfully translated into clinical practice. One of the key challenges is the incomplete understanding of how the different PCD pathways interact with each other. For instance, LPO, a hallmark of ferroptosis, may inadvertently suppress pyroptosis by altering membrane fluidity or sequestering essential mediators like GSDMs. Additionally, the role of necroptosis in modulating T cell exhaustion or myeloid cell polarization remains unclear, complicating the rational design of combination therapies. Furthermore, most preclinical studies rely on murine models or cell lines that do not fully capture the genetic diversity or immunosuppressive microenvironments of human melanoma, limiting the predictability and relevance of these findings. The lack of standardized models for therapy-resistant niches, such as brain metastases, further complicates the translation of preclinical data into clinical success.

Another significant hurdle lies in the translation of nanotherapy from preclinical studies to clinical use. While nanotechnology platforms, including MOFs and lipid-coated nanoparticles, have shown promising results in preclinical studies (229–232), their clinical application faces challenges such as variability in batch production, poor tumor penetration in desmoplastic tissues, and potential toxicity from metal ion leakage. Additionally, the absence of scalable manufacturing processes for these complex nanocarriers raises concerns regarding cost-effectiveness and regulatory approval. Moreover, current biomarkers, such as PD-L1 expression and TMB, have demonstrated limited predictive value for immunogenic therapies, highlighting the need for the development of more precise biomarkers to guide treatment selection. For example, patients with high PScore may benefit from ICD-inducing agents, but no validated assays currently exist to guide such stratification. The influence of factors such as gut microbiota, systemic metabolism, and host genetics on therapy outcomes remains underexplored, contributing to inconsistent results in clinical trials.

## 5 Technological and preclinical frontiers

Addressing these complex questions requires sophisticated tools. Cutting-edge technologies such as single-cell omics and spatial transcriptomics have offered an in-depth understanding of melanoma’s heterogeneity and its immune landscape. These approaches have revealed rare subpopulations of therapy-resistant melanoma cells and highlighted the dynamic shifts in immune cell states during treatment. This high-resolution analysis, powered by computational methods like BayesSpace for clustering spatial data, has facilitated the design of precision therapies aimed at vulnerabilities in both malignant and stromal compartments. However, a key challenge remains in integrating multi-modal data to enhance resolution, as spatial transcriptomics alone can sometimes lack single-cell precision.

Artificial intelligence (AI) and machine learning (ML) are also poised to play a transformative role. AI/ML algorithms can be applied to analyze vast datasets to predict the efficacy of novel drug combinations, identify new therapeutic targets, and optimize treatment regimens. For example, neural networks can be trained to predict cell viability and dose-response curves for complex combination therapies, guiding preclinical development. This computational power will be essential for navigating the immense complexity of targeting multiple RCD pathways simultaneously in heterogeneous patient populations.

## 6 Future directions and the path to clinical translation

The journey from compelling preclinical concepts to effective clinical therapies is fraught with challenges that must be systematically addressed.

### 6.1 Developing predictive biomarkers and optimizing clinical trials

A significant barrier to clinical translation is the lack of biomarker panels to monitor patient response to therapies targeting multiple RCD pathways. Current clinical protocols for melanoma incorporate biomarkers for targeted therapy (e.g., BRAF mutation status) and immunotherapy (e.g., PD-L1 expression) but none are established for this novel approach. Developing assays to measure the activation state of ferroptosis, pyroptosis, and necroptosis within tumors is a critical first step for personalizing treatment and assessing therapeutic efficacy.

Furthermore, clinical trial designs must be adapted to evaluate these complex combination strategies. Traditional linear trial designs may be inadequate. Instead, adaptive trial designs, such as Sequential, Multiple Assignment, Randomized Trial (SMART) designs, will be necessary to flexibly test different combinations, doses, and schedules based on emerging data, thereby optimizing the path to regulatory approval.

### 6.2 Overcoming manufacturing and delivery hurdles

For strategies involving multifunctional nanoparticles, significant translational barriers exist, including challenges in manufacturing at scale, ensuring batch-to-batch consistency, predicting and managing toxicity, and overcoming biological barriers to ensure effective tumor delivery. The gap between promising results in preclinical animal models and outcomes in human patients remains a major hurdle for nanomedicine in general.

### 6.3 Expanding the mechanistic framework to include the microbiome

The gut microbiome has emerged as a critical modulator of response to immunotherapy in melanoma patients. However, its role in modulating the response to therapies that induce ferroptosis, pyroptosis, and necroptosis is entirely unknown. Future research must investigate whether the microbiome influences the activation of these RCD pathways or the subsequent immune response, potentially opening avenues for microbiome-based co-therapies to enhance treatment efficacy (233, 234).

## 7 Conclusion

The strategy of targeting ferroptosis, pyroptosis, and necroptosis in concert represents a paradigm shift in cancer immunotherapy for melanoma. It moves beyond single-target approaches to embrace the complexity of tumor biology, aiming to induce a multi-faceted, immunologically potent form of cell death that can overcome intrinsic and acquired resistance. While the preclinical rationale is strong and supported by emerging evidence, the path forward requires a concerted, interdisciplinary effort. Key priorities must include elucidating the intricate molecular cross-talk between these pathways, developing robust biomarkers for patient selection and response monitoring, and designing innovative clinical trials capable of navigating the complexities of combination therapies. By addressing these fundamental challenges, the immense therapeutic potential of this triad approach may be unlocked, offering new hope for patients with advanced melanoma.

## Glossary

IL-2: interleukin-2
ICIs: immune checkpoint inhibitors
BRAF: B-Raf proto-oncogene, serine/threonine kinase
MEK: Mitogen-Activated Protein Kinase
CTLA-4: Cytotoxic T-Lymphocyte Antigen 4
TMB: tumor mutational burden
irAEs: immune-related adverse events
PCD: Programmed cell death
DAMPs: damage-associated molecular patterns
DCs: dendritic cells
CTLs: cytotoxic T lymphocytes
RIPK1: Receptor-Interacting Protein Kinase 1
HMGB1: High Mobility Group Box 1
ICD: immunogenic cell death
TME: tumor microenvironment
Tregs: regulatory T cells
MDSCs: myeloid-derived suppressor cells
RCD: Regulated Cell Death
TFRC: transferrin receptor
ROS: reactive oxygen species
ALOXs: arachidonate lipoxygenases
PUFAs: polyunsaturated fatty acids
PL-PUFA-OOH: phospholipid hydroperoxides
ACSL4: Acyl-CoA Synthetase Long Chain Family Member 4
LPCAT3: Lysophosphatidylcholine Acyltransferase 3
GSH: glutathione
PDAC: pancreatic ductal adenocarcinoma
cTRIP12: circTRIP12
OGT: O-GlcNAc transferase
FTH: ferritin heavy chain
KEAP1: Kelch-like ECH-associated protein 1
SCLC: small cell lung cancer
GBM: glioblastoma
HCC: Hepatocellular carcinoma
MLKL: mixed-lineage kinase domain-like
CYLD: Cylindromatosis
ZBP1: Z-DNA Binding Protein 1
EMT: epithelial-mesenchymal transition
HNSCC: head and neck squamous cell carcinoma
NSCLC: non-small cell lung cancer
GSDM: gasdermin
GSDMD: Gasdermin D
NK: natural killer
ICB: immune checkpoint blockade
TNBC: triple-negative breast cancer
dsRNA: double-stranded RNA
RIDD: regulated IRE1-dependent decay
GC: gastric cancer
NLRP3: NLR Family Pyrin Domain Containing 3
SLC7A11: Solute Carrier Family 7 Member 11
Gpx4: GSH peroxidase 4
ART: artemisinin
FFAs: free fatty acids
CAMKK2: Calcium/Calmodulin-Dependent Protein Kinase Kinase 2
zVAD-fmk: Z-Val-Ala-Asp(OMe)-FMK
IFNγ: interferon-γ
ADAR1: Adenosine Deaminase Acting on RNA 1
ZBP1: Z-DNA Binding Protein 1
ISGs: interferon-stimulated genes
HMOX1: heme oxygenase 1
PScore: pyroptosis score
CRKL: C-reaK-tyrosine kinAse like
SOX10: (Sex Determining Region Y)-box 10
Clo: clofarabine
R@AZOH: R848-loaded AlZn hydroxide
RNS: reactive nitrogen species
BRAFi: BRAF inhibitors
MEKi: MEK inhibitors
CDNP: Cyclic di-nucleotide phosphodiesterase
TAMs: tumor-associated macrophages
PRGs: pyroptosis-related genes
MOF: metal-organic framework
LPO: lipid peroxidation
scRNA-seq: single-cell RNA sequencing
GEMM: genetically engineered mouse model
IFN: interferon
ISGs: IFN-stimulated genes
ECM: extracellular matrix
FATP: fatty acid transport protein
TANs: tumor-associated neutrophils
TILs: tumor-infiltrating lymphocytes
